# Cascading training the trainers in ophthalmology across Eastern, Central and Southern Africa

**DOI:** 10.1186/s12992-017-0269-x

**Published:** 2017-07-10

**Authors:** Melanie C Corbett, Wanjiku Mathenge, Marcia Zondervan, Nick Astbury

**Affiliations:** 1 The Western Eye Hospital, Imperial College NHS Trust, 153-173 Marylebone Road, London, NW1 5QH UK; 2 Royal College of Ophthalmologists (RCOphth), 18 Stephenson Way, Euston, London, NW1 2HD UK; 3 Rwanda International Institute of Ophthalmology, Dr Agarwal’s Eye Hospital, 19 KG 201 St, PO BOX 312, Kigali, Rwanda; 4 College of Ophthalmology of Eastern Central and Southern Africa (COECSA), Regent Court, Block A, Suite A7, Argwings Kodhek Road, Hurlingham, PO Box 4539, 00506 Nairobi, Kenya; 5 International Centre for Evidence in Disability, London School of Hygiene & Tropical Medicine, Keppel Street, London, WC1E 7HT UK

**Keywords:** Trainers, Training, Teaching, Supervision, Human resources, Partnership, Sustainability, Ophthalmology, Sub-Saharan Africa, VISION 2020

## Abstract

**Background:**

The Royal College of Ophthalmologists (RCOphth) and the College of Ophthalmology of Eastern Central and Southern Africa (COECSA) are collaborating to cascade a Training the Trainers (TTT) Programme across the COECSA Region. Within the VISION 2020 Links Programme, it aims to develop a skilled motivated workforce who can deliver high quality eye care. It will train a lead, faculty member and facilitator in 8 countries, who can cascade the programme to local trainers.

**Methods:**

In phase 1 (2013/14) two 3-day courses were run for 16/17 selected delegates, by 3 UK Faculty. In phase 2 (2015/16) 1 UK Faculty Member ran 3 shorter courses, associated with COECSA events (Congress and Examination).

A COECSA Lead was appointed after the first course, and selected delegates were promoted as Facilitators then Faculty Members on successive courses. They were given appropriate materials, preparation, training and mentoring.

**Results:**

In 4 years the programme has trained 87 delegates, including 1 COECSA Lead, 4 Faculty Members and 7 Facilitators. Delegate feedback on the course was very good and Faculty were impressed with the progress made by delegates.

A questionnaire completed by delegates after 6–42 months demonstrated how successfully they were implementing new skills in teaching and supervision.

The impact was assessed using the number of eye-care workers that delegates had trained, and the number of patients seen by those workers each year. The figures suggested that approaching 1 million patients per year were treated by eye-care workers who had benefited from training delivered by those who had been on the courses.

Development of the Programme in Africa initially followed the UK model, but the need to address more extensive challenges overseas, stimulated new ideas for the UK courses.

**Conclusions:**

The Programme has developed a pyramid of trainers capable of cascading knowledge, skills and teaching in training with RCOphth support. The third phase will extend the number of facilitators and faculty, develop on-line preparatory and teaching materials, and design training processes and tools for its assessment. The final phase will see local cascade of the TTT Programme in all 8 countries, and sustainability as UK support is withdrawn.

## Background

Sub-Saharan Africa bears a significant burden of visual impairment (21.4 million people) and blindness (4.8 million people) [1, 2]. This is paralleled by a huge shortage of ophthalmologists (3.1 per million population) [3, 4]. The aims of this study were two-fold: firstly to describe and evaluate a Health Partnership Model of human resource development that would be self-sustaining beyond partnership involvement; and secondly, to assess the potential breadth of impact of the model on the delivery of eye care. It also describes an effective model of up-skilling trainers that can be applied to any specialty or profession in healthcare systems across the world.

The imbalance between eye-care demand and service capacity has been targeted by “VISION 2020: The Right to Sight”, a global initiative established through the partnership of the World Health Organization (WHO) and the International Agency for the Prevention of Blindness (IAPB). It has set targets for the reduction of avoidable visual impairment by the year 2020, and is dependent upon huge developments in Human Resources for Eye Health (HReH) [4, 5].

### The VISION 2020 LINKS programme

Human resources development is the focus of The VISION 2020 LINKS Programme, which was established in 2004 through the International Centre for Eye Health (ICEH). It matches an African eye department with a UK eye department in a partnership to train the whole eye care team [6].

Within this programme in 2009, a LINK was established between the newly established Eastern African College of Ophthalmologists (EACO) and the Royal College of Ophthalmologists [7]. EACO represented three countries, and wanted help to set up and run a College-based exam. 6 years later it expanded to become The College of Ophthalmology of Eastern Central and Southern Africa (COECSA) including 9 countries, and recently a further 3 have joined [8]. When considering further expansion, COECSA is currently keen to concentrate on those countries with a good level of English amongst its ophthalmologists.

A 2 year grant from the Tropical Health Education Trust (THET) Health Partnerships Scheme [9, 10] funded the implementation of five projects by the COECSA-RCOphth LINK to help set standards in clinical practice and education, and to develop human resources in training and leadership (Guidelines & Continuing Professional Development, Curriculum, Examinations, Leadership and Training the Trainers). The Training the Trainers (TTT) Programme was modeled on the successful UK programme and adapted to the African environment.

### Ophthalmology training in Eastern, Central and Southern Africa

The provision of ophthalmology training throughout the COECSA Region is variable because it is largely managed and delivered by individual universities, of which there are mostly one or two per country. Each sets its own standards for its local curriculum and exams, and there is minimal quality assurance of trainers. The 3–4 year training is delivered largely by university teachers, with some elective time spent in peripheral units.

Much of the teaching is didactic in a class room situation with relatively large student:teacher ratios. There is a heavier emphasis on the acquisition of knowledge and comprehension (the lower levels on the pyramid of learning), and less on the application of that knowledge to analyse, make judgements, problem solve and evaluate (the higher levels on the learning pyramid). In the UK these higher skills are largely taught during clinical work with patients, and a relatively low student:teacher ratio.

In general, there is relatively little educational supervision of trainees. The good trainees will succeed through self-motivation, but there is relatively little pastoral care of those who are struggling, whether it be for work-related or personal reasons. Large cohorts of trainees pass through a training programme that is relatively standardised for their institution, without much tailoring for their individual training needs or personal situation.

### The UK model of medical education and training

Medical Training in the UK has evolved rapidly over the last 20 years [11]. We have left behind a system where progress through a haphazard collection of jobs was largely determined by examination results and recruitment interviews. Nowadays progress through a structured national training scheme is determined by robust assessments of all areas of medical practice made against rigorous standards set in a detailed curriculum. The whole process is overseen by the General Medical Council (GMC) and results in a Certificate of Completion of Training (CCT) or equivalent. Trainees are heavily supported by a raft of supervisors in everyday clinical situations, and overall through regular educational appraisals and reviews. Trainers now have to demonstrate that they keep their skills up-to-date in the seven domains of the GMC’s National Framework for Trainers [12].

### Training the trainers in ophthalmology in the UK

The Royal College of Ophthalmologists (RCOphth) established its Training The Trainers (TTT) Programme in 2005. At this time teaching and training were emerging as important skills that could be improved with formal training. This required some doctors to become specialists in Medical Education and Training. The RCOphth initially delivered an intermediate level course across three individual days. Since then this course has evolved, keeping pace with, and in some areas leading, new skills and processes in education and training. Further courses have been designed at Trainee and Advanced Trainer levels to address TTT needs at all parts of the trainer’s career.

### The COECSA-RCOphth TTT partnership

The COECSA-RCOphth TTT Partnership was set up in 2013 and has an 8 year plan (in four 2-year phases) coinciding with the remaining time frame of the overall VISION 2020 Project. It aims to role out a TTT Programme across the COECSA region to develop a skilled motivated workforce functioning to their full potential to deliver high quality eye care.

**Fig. 1:**
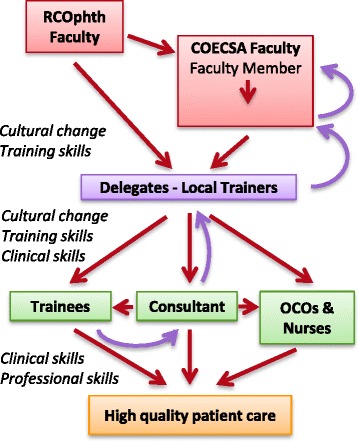
The TTT Cascade. *Red arrows* show the cascade of the training ethos, knowledge and skills from faculty to delegates to local healthcare workers. The *purple arrows* show the development of staff, with trainers progressing to facilitators then faculty members

Its success depends upon a cascade of skills though adequately trained tiers of individuals, from COECSA level, through national level to the institutions or units. Delegates who show potential on the courses are promoted through the ranks of Facilitator, where they assist with practical sessions on the next course, to Faculty Member when they can lead the delivery of material, as can the COECSA Lead (Fig. 1).

The aim is to train a National TTT Lead, a Faculty Member and a Facilitator in each of the 8 countries, who can cascade the programme to local trainers. In due course this could extend beyond medical graduates to the Ophthalmic Clinical Officers (OCOs).

By the end of 2016 the first two phases of the programme were complete and funding had been obtained for the next three years (2017–2019) through the Commonwealth Eye Health Consortium (CEHC) funded by the Queen Elizabeth Diamond Jubilee Trust (QEDJT). By the final year, 2020, the process should be self-sustaining.

The other stakeholders in this area include Orbis, The International Council of Ophthalmology (ICO) and the VISION 2020 LINKS in individual hospitals. Working together will help ensure an integrated approach at both regional and institutional levels to optimize outcomes.

## Methods

This study describes how the TTT project was set up, using a model that can be applied to other specialties and locations. It identifies the features that are most important to consider when implementing such a model, and evaluates its potential breadth of impact.

The TTT Programme is funded through peer reviewed grants (2013–14 through THET; 2017–19 through CEHC and QEDJT). Ethics committee approval was not required as the methodologies used were description, monitoring and evaluation of the programme.

### Formation of the partnership

At the time the partnership was set up between the two Colleges, there were existing successful links between institutions in Africa and the UK. When the new Eastern African College of Ophthalmology was set up, it was appropriate that it should turn to the UK College for partnership as their overall structure and goals would be well-matched. The first projects taken on were very specific with finite end points: to design a curriculum, and to set up a fellowship examination. This paved the way for more complex projects in leadership and training.

### The start of the TTT project – the first course

The TTT Project started in 2013 with three RCOphth Faculty members (including the UK TTT Lead) running a 3-day TTT Course in Kenya for delegates comprising two senior educationalists from each of the 8 countries. English is the primary professional language in 7 of the countries. Delegates from Burundi had an adequate level of understanding and expression in English, despite their primary professional language being French.

For the first course it was important to have invited delegates responsible for the management of training, because any subsequent changes to training would require their understanding and cooperation. The second course had a different group of delegates (apart from the 3 promoted to be facilitators), who were more actively involved in delivering the training. They tended to be enthusiastic and open-minded, and had more direct contact with the trainees.

During the first course, time for discussion was taken both during the teaching itself and informally during breaks, for the UK Faculty to understand the training ethos and processes in the host countries, and the objectives of the delegates. Training material was adapted accordingly during the course.

### Aims of the TTT project

**Fig. 2:**
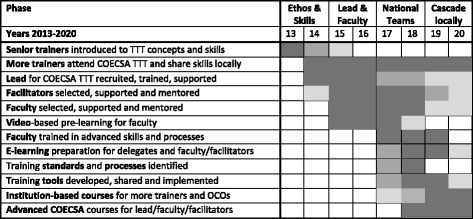
Project plan for the COECSA-RCOphth TTT Programme. The 8 year programme is divided into four phases which build national faculty teams who will be able to deliver the training locally. OCO = Ophthalmic Clinical Officer

It was only after the first course that the detail of the long-term aims and delivery of the project could be defined. The COECSA-RCOphth TTT Programme spans 8 years, delivered in four phases, each of two years duration (Fig. 2). The aims of the project by 2020 are to achieve:

- Establishment of an ethos of training that embeds the attitudes and skills taught on the course, within the training programmes of all institutions (2013–14).
- A Lead for the COECSA TTT Programme who is responsible for coordinating the whole programme and representing TTT within COECSA (2015–16).
- A team of 1 national lead, 1 faculty member and 1 facilitator per country who can role out courses and support trainers nationally (2017–18). They will develop their skills through the COECSA Advanced TTT Courses (2019).
- More than 50% of trainers to have received at least basic level TTT training in supervision and implemented the skills into their practice (2020).

### The second course and development of facilitators

In 2014 a second 3-day course was delivered in a similar way to the first course. Different delegates were invited from across the 8 countries, with the exception of three from the first course who were invited back to be facilitators, one of whom was identified as the COECSA TTT Lead. These facilitators were sent a slide set for the lecture they were asked to deliver, a pre-recorded video of that lecture from which they could learn, and the instructions and material for running the associated practical session.

During the course each African Facilitator was paired with a UK Faculty member who mentored them in the preparation of their material. At the end of the course there was a meeting for the six Faculty and Facilitators. During the first half of the meeting they all received constructive personal feedback from each other on their contributions. The second half was a structured discussion resulting in a forward plan for the further development of the project.

### Continuing the project on minimal funding

After the first two years, there was minimal further funding with which to continue the project. The delegates were at a stage when they had some new training skills, but not the ability roll out the rest of the project. Three further courses were run by the UK TTT Lead supported by African facilitators for delegates already attending another event. In 2015 a half-day symposium preceded the COECSA Congress, and a 1-day course for examiners was run the day before the examination. During the examination the UK TTT Lead observed the examiners and gave personalized feedback on their communication and examination skills. In 2016 a 1-day symposium lead by the UK TTT Lead was part of the Annual Congress.

### Design of the TTT courses

The courses concentrate on the learning, practice and implementation of the skills required for teaching and training. These are underpinned by a limited amount of theoretical knowledge where necessary [13, 14].

The 3-day courses are based on the Supervisor level courses run in the UK. They aim to create a group of ophthalmologist who can:

- Teach clinical skills effectively
- Develop individual healthcare workers through supervision
- Manage training programmes
- Cascade training skills to others

The course is delivered through a mixture of didactic/discursive material and practical skills. In the UK, delegates are given pre-learning in which they independently study pre-recorded lectures and complete reflective work before attending the course [15]. This enables them to understand the tools to be used and how to apply them. In Africa this was trialed with the first facilitators, but only used to a limited extent thereafter because the material requires more specifically tailored explanations than can be delivered in a pre-recorded format. Therefore the material is delivered during the course, but in an interactive manner. DVDs have been made which can be sent to facilitators on future occasions, as they already have an understanding of the material.

During the first session of a 3-day course the delegates and faculty are asked to introduce themselves and state one of their key objectives for the course. This enables the group to get to know each other, sets an interactive mode, enables everyone to contribute early and informs the faculty about the delegates’ priorities and vice versa.

For over half the time on the course, delegates take part in multiple intensively-facilitated small group discussions, practical sessions and role plays. These give them the opportunity to fine-tune their skills through practice, observing others and receiving personal feedback.

The consolidation phase of each course requires all the delegates (including facilitators and faculty) to decide, write down and discuss a personal development plan (PDP) for themselves, their unit and their country, at each of the time points 2 weeks, 2 months and 2 years. These were shared with the group, and areas identified that others might want to add to their PDP, or where collaboration between delegates would help. They also helped the Faculty identify priorities for future courses and input.

### Content of the TTT courses

The three day courses are delivered in three modules (Table 1). The shorter courses concentrate on the key basic skills from the first two days, such as setting aims & objectives, designing a teaching session, feedback and appraisal. It is most important that trainers enhance trainees’ learning by developing their repertoire of teaching techniques, and practicing their conversation skills used during feedback and supervision.

**Table 1:** Course objectives for delegates

Module Objectives	Teaching & Enhancing Learning	Supervision in Practice	Programme management & Trainees in difficulty
Didactic/discursive material Delivered through pre-learning or interactive lectures *Objective:* Understand the tools and how to apply them	Techniques to enhance the value of teaching Tailoring teaching to the learners Setting aims and objectives Designing a teaching session Teaching practical skills, including surgery [13] Evaluation and feedback	Feedback skills [14] Appraisal skills Curriculum implementation Formative assessment by WBAs and portfolio Summative assessment Assessment meetings with trainees Trainer behaviours	Spectrum of TiDs Difficult feedback Complex appraisals Using objective assessment in target setting Stages and techniques for managing TiDs Recruitment Rotations Trainer in difficulty Unit in difficulty
Practical skills Including multiple, intensively-facilitated small group discussions, practical skills and role plays *Objective:* Fine tune their skills through observation, practice and individual feedback	Use objective setting to stimulate and direct learning Plan a teaching half day to maximise interest and learning Deliver a lecture Use interactive techniques Use a four-step approach to teaching practical skills [13] Evaluate teaching and improve through feedback [14]	Use objective setting to stimulate and direct learning Motivate trainees through feedback [14] Improve learning from formative assessment Make accurate summative assessments for supervisor reports Develop action plans through which to monitor progress Guide personal and professional development Talk to trainees about their shortcomings Use questions to solve problems	Recognise a trainee in difficulty Instigate mechanisms for early detection Collect appropriate evidence Discuss problems with a trainee and assess insight Set an action plan Write an objective report Access help and support

The course is delivered in three modules which each have didactic/discursive and practical components

*WBA* Work-place Based Assessment, *TiD* Trainee in Difficulty

### Evaluation of the courses

Immediately after all five courses delegates were asked to complete a course evaluation form comprising grades for the quality of each section, and free text comments on the course and ideas for future developments. After the two 3-day courses, feedback was also collected from African Facilitators and the UK Faculty.

### Evaluation of the implementation of the TTT skills

The existing level of delegates’ training skills were informally assessed by the UK Faculty at the start and during the course from discussions and whilst observing practical skills.

All delegates attending 3-day courses were sent a questionnaire 6 months to 3.5 years after their first course to ascertain their implementation of the skills they had learnt. For skills where the quality was to be assessed, delegates were asked how often they performed them in a particular way (grades from “never” to “always”). For processes that could be utilized or not, delegates were asked how often they performed them in 3 months (graded 0 to >6).

### Evaluation of the impact of the TTT programme

The questionnaire also collected data on the estimated number of healthcare workers to whom the delegates taught clinical skills or training skills. It asked them to estimate the approximate number of patients seen by these individuals, as a proportion of the total of all the patients seen in their hospital per year. This was used to calculate the potential number of patients who could have been managed by an individual benefitting from the course.

## Results

17 and 16 delegates attended the first and second 3-day courses, with the three African Facilitators on the second course having attended the first course. 26 examiners attended a one-day course in 2015, and 48 delegates attended a half- or one-day course at the COECSA Congress in 2015, 2016 or both. The database of TTT Participants now contains 87 delegates, within which there is so far one COECSA Lead, 4 Faculty Members and 7 Facilitators. Further delegates from each country have been identified for promotion to each level on future courses.

### Adaption of the courses for the COECSA delegates

On the first course in particular, it was vital to rapidly ascertain how training material and approaches needed to be adapted for the new audience, and for the faculty to remain flexible throughout the course.

It was important to identify how the two training structures equated, and to then use their local terminology where it existed, for example: “university” instead of “deanery”. Where no equivalent existed, for example “Clinical Supervisor” and “Educational Supervisor” it was necessary to define the terminology at first use, but then on subsequent use, give a reminder of which is being discussed, for example “the Educational Supervisor, who is responsible for the overall progress of the trainee”.

Abbreviations could be used on the slides, as long as a longhand form was said when pointing to the abbreviation, for example, ARCP is referred to as “Annual Review” without having to repeat “of Competency Progression” on each occasion. References to time in training had to be adjusted pro rata for the average time taken in Africa, for example a “Year 7 trainee” became a “Year 4 trainee”.

When suggesting changes in behaviour it is important to show understanding of why their current practice is the way it is, without the delegates feeling inadequate or belittled. Cultural difference have to be treated sensitively, such as a greater respect for hierarchy, a lesser appreciation of the requirement to nurture trainees in difficulty, or the need for the removal of external influences from a fair and transparent assessment process.

Role play is used at various points in the course for learning and practicing new skills, behaviours and conversation techniques, for example conducting an appraisal and giving feedback. The scenarios have to be as life like as possible, so it is valuable to discuss them with one of the delegates beforehand and modify them accordingly. The whole concept of role play was totally new to the delegates, as became obvious when they did not know how to approach the first scenario. Once the UK faculty had demonstrated one role play scenario, they rapidly learnt what they had to do, and were very effective at improving their skills.

### Evaluation of the courses

Written feedback from all delegates on the lectures included “excellent”, and “very practical, interesting and engaging”, with the majority of sessions being scored “very good” (highest category) by over half the delegates, and the remainder scoring “good”. This was also the case for the sessions delivered by African faculty, although their results were not quite as high as for the UK faculty.

The delegates appreciated learning “how to be systematic” in their approach to training, and to “have a good feel about being a trainer”. They found the Supervision sessions including feedback most valuable because they are “activities we should carry out regularly” and “have tremendous impact on the performance of a trainee”.

Half the course was practical skills, and these components were very highly rated, with the majority of delegates scoring them the highest category, “very good”. Delegates found that the practical sessions “helped them to internalise the lectures, because once you do something it is easier to recall”. The feedback received during practical sessions was very useful because “gave ideas for improvement, even for good students”. Of particular value was appraisal, teaching practical skills and the trainee in difficulty, because they showed that “what is the best way depends on the circumstances, which calls for flexibility”. They found the role plays valuable because “practicing trainer on trainer, you observe a unique experience, and have all the feelings in a shorter time”. Suggestions for next time included requests for shorter lectures and an even greater practical component to the course.

The delegates concluded that it “helped us reflect on what we are doing, evaluate ourselves and set plans for improvement in delivering teaching, training and supervision”. “This very important programme should be embraced all over the region, locally and nationally”.

### Delegate action plans

Items from delegates’ personal action plans were combined to facilitate development of the group and also the TTT Programme as a whole (Table 2).

**Table 2:** Items from Delegate action plans drawn up at the end of the second 3-day course

Time scale	Action Plan for the individual/unit/country
2 weeks	give feedback using the four step technique [14] perform an appraisal with at least one trainee discuss learning from the course with local trainers discuss progress with one other delegate by telephone enter each action point onto an personal development plan listing: objective, action required and outcome/evaluation, all with dates COECSA Administrator to coordinate circulating e-mails to the group to keep them in touch regularly.
2 months	write aims and objectives for their own existing lectures and rework lectures to address these plan teaching sessions to incorporate a variety of styles and techniques, including preparation by audience and increasing interaction use the four-step technique for teaching practical skills [13] apply teaching methodologies to all professions, eg: including OCOs nurses, to roll out a change in ethos use evaluation forms to collect feedback on teaching create a comfortable learning environment in theatres, including storing the vitrector in the theatre for ease of access and communication communicate what has been learned on the course to the rest of the department, and work out how to get them on board, possibly using existing departmental meetings incorporate teaching on training into existing teaching sessions review current training systems in the unit and identify which parts would be easiest to modify first allocate an educational supervisor to each trainee, and ensure they know they have a role in the career development of those trainees introduce a trainee portfolio develop an appraisal system for trainees with the necessary tools, and communicate its implementation to trainers and trainees
2 years	RCOphth is helping design the curriculum with WBAs to include in the trainee portfolio develop an appraisal system for trainers evaluate the quality of the training programme and list areas for improvement evaluate specific training areas, eg: cataract outcomes use evidence collected to influence any difficulties in cascading which may occur at country level design methods for cascading Training the Trainers Ophthalmic Committee Meetings for COECSA or Ophthalmic Societies to be used as a focus for further Training the Trainers sessions COECSA Annual Conference to have a session on training each year

Delegates were asked to write down actions at individual, unit and COECSA levels, which they wanted to undertake by 2 weeks, 2 months and 2 years

*OCO* Ophthalmic Clinical Officer*, WBA* Work-place Based Assessment

### Feedback from African facilitators

The three COECSA Facilitators on the second course found the experience both “exciting” and “challenging”, and it gave them “glimpses of the excellence” they hope to achieve. They obtained a “huge learning experience as teachers” and benefitted from the feedback and mentoring they received. The course extended their thinking because it “highlighted areas for development for both trainer and trainee” and discussed “new processes that they had not experienced as students”. The course itself was valuable to them as faculty members because it “gave a structure to guide teaching and training” and utilized “well defined mechanisms for influencing change”.

The COECSA Faculty felt that although they had learnt a lot from the two courses they had attended, it was “too early for them to be entrusted with running the course” themselves. They would need “more than one exposure to the course” as a facilitator, and the support of a “forum to pass on the learning”. They would then feel more comfortable “facilitating in their own region” before leading an international course.

### Feedback from the RCOphth faculty

The faculty found the delegates very engaged with the course, and committed to completing the exercises outside the course, and taking part in the practical activities. There were certain areas where the delegates had limited prior knowledge, such as having conversations with trainees for appraisals or managing the trainee in difficulty. However, once a role play was demonstrated, they rapidly picked up the new skills. The three COECSA Faculty members on the second course were seen to be very committed to their roles.

### Implementation of training skills

Observations by the UK Faculty of delegates’ knowledge and performance at the beginning of discussions and practical skills concluded that delegates had limited prior knowledge or experience of many of the specific techniques taught on the course, or training processes akin those used in the UK. These were the areas targeted for determination of whether skills had been successfully implemented since the course or not.

Implementation was best for teaching skills and simple supervision skills by the individual. Fewer delegates had managed to implement more complex supervision skills, or aspects of training that required involvement of colleagues or changes to training processes (Table 3).

**Table 3:** Implementation of the TTT Programme

Implementation	Skills successfully implemented	Skills yet to be implemented
Success = performed by ≥50% of delegates “often” or “always”	write aims and objectives for teaching use a variety of teaching styles use interactive techniques in teaching use 4-step technique for practicals give feedback after supervision use 4-step technique for feedback ensure trainees have educational supervisor	obtain formal evaluation of teaching sessions give feedback after supervising someone of another profession
Success = performed by ≥50% of delegates at least 4 times in 3 months	deliver a formal teaching session supervise a trainee or others give formal feedback / appraisal formally assess a trainee	appraise a trainee appraise a consultant colleague or someone from another profession formally assess a trainee (eg: work place based assessments) have responsibility for a trainee in difficulty (TiD) develop other trainers (eg: lectures, planning meetings, formal discussions)
Success = performed by ≥50% of delegates once in 3 months	review and update your personal development plan (PDP)	

Skills were considered to have been successfully implemented if they were performed by over half of delegates (*n* = 20) with an appropriate frequency at the time of the follow up questionnaire

### Impact of the TTT programme

Estimates made by delegates of the number of people affected by the course at each stage of the cascade gives some idea of the magnitude of the potential impact of such a programme.

The 30 delegates attending the first two courses trained an average of 22 eye care workers in clinical skills and 8 in training skills. They worked in a total of 20 different hospitals which saw an average of 44,000 patients per year. The delegates estimated that 70% (on average) of those patients were seen by a healthcare professional who had received some benefit from the TTT course. Therefore about 620,000 patients per year (20 × 0.7 × 44,000) could have been seen by an eye care worker whose training could have been improved by the course. The delegates also helped train 14 eye care workers (on average) from other hospitals, which means that the over all impact of the first two courses could be affecting the care of approaching 1 million patients per year.

## Discussion

Improvement in the vision of patients across Sub-Saharan Africa is dependent upon developing a skilled effective workforce delivering high-quality eye care to their full potential [3]. This requires unified standards set by curricular frameworks for trainees, trainers and units which are assessed using objective tools [11, 12]. The effective learning of knowledge, skills, attitudes and behaviours, across both the clinical and professional spectrums, needs to occur in a supportive training environment which encourages every individual to flourish.

The evolution of such a training system in the COECSA Region started with the insight of a few individuals that there could be a better training system, and that external help would be required to support its introduction. Once the partnership was set up, the programme passed through the overlapping phases of changing ethos, and developing training skills in faculty and delegates. It is now ready to tailor the necessary training processes and tools to the African environment, so the faculty can start a full cascade of these tools and skills in each country. Within a few years the programme should be self-supporting and fully sustainable.

### Setting up the COECSA-RCOphth partnership

The link between COECSA and RCOphth has proved to be an excellent partnership, through achieving many of the essential principles proposed by THET [16, 17]. At the birth of the fledgling EACO (precursor of COECSA) the match looked sensible due to both Colleges being membership organisations and possessing similar values and goals. RCOphth had a commitment to overseas development as stated in its charter [18].

Such a partnership was well-aligned with the aims of VISION 2020, which had already had success twinning African institutions with those in the UK. It was therefore appropriate for this partnership to operate under the auspices of VISION 2020, and benefit from their experience in the field [5, 6].

### Setting up the TTT programme

Prior to the TTT Programme, the two colleges had already worked together to establish both a curriculum and an examination for COECSA [7]. These set the educational standards which provided the motivation for introducing a robust training system and developing the necessary trainer skills.

RCOphth ran a successful TTT Programme in the UK, and had international experience by delivering single courses to Egyptian and Irish ophthalmologists. It was understood that the programme would need to be modified for Africa, but it was difficult to determine exactly how that would be, prior to meeting the delegates.

### Initiating the TTT programme

The UK faculty attending the first course were highly experienced trainers, and were very familiar with delivering the course in the UK. They took with them a wide range of materials which were adapted to meet the delegates’ needs. At the beginning of the course the variation in training delivery around the region was ascertained, and the overall level of training knowledge and experience of the delegates was informally assessed during the discussions. In one of the early sessions they were each asked to share with the group one of the main objectives they had for the course.

The delegates selected to attend the first course were two of the senior educators from each country. It was important for them to be on board with the change in ethos required and inputting into what needed to be done, even if subsequently it was to be some of the other trainers who implemented the processes and rolled out the skills training.

The aim of the first course was for the delegates to become aware of the concepts central to effective training and some of the skills required. A change in ethos was required before any new training techniques could be implemented. It was noticeable even by the second course that the ethos of the delegates was more aligned to those of the UK faculty, for example that teaching should be more interactive, and that Trainees in Difficulty (TiDs) required help and support.

It is important that the goals in each stage of the programme are achievable, in order to build mutual trust, and so that participants feel that progress is being made [16, 17]. This is particularly important early on as subsequent goals can then be directed by the needs identified during the first phase and the speed of progress. This was why the objectives of the programme as a whole were not drawn up until after the first course, and were adapted over the years.

### Design of the TTT course

The course concentrates on the learning and implementation of training skills, and includes theory only where necessary to support this [15]. Delegates valued the large proportion of time spent on practical skills so they could observe others, practice their skills and receive personalised constructive feedback. This technique is of particular importance when facing the difficult task of changing behaviour. In the UK delegates can go straight into role play practicals, but the Africans new to the course were unfamiliar with the concept and required a demonstration beforehand. However, once they understood what was required, most could develop the skills rapidly.

In the UK, the knowledge-based portion of the course is delivered through preparatory work in the form of videoed lectures, with good uptake [15]. Few African delegates accessed these as they were not used to doing preparation for courses. The three African faculty attempted to access the lecture they were due to deliver, but found the streaming frustratingly slow. In subsequent courses, minimal pre-learning was set, and it was in the form of reflective writing on downloadable templates. The uptake was still small, although it would probably be better for those who had attended a previous course and therefore understood its value. The TTT Course in the UK is moving to an e-learning format for pre-learning, which will also be available overseas.

The introductions and sharing of objectives at the beginning of the course are valuable in setting an interactive tone, and ensuring that the course is maximally effective for the delegates [16, 17]. This session is particularly valuable for the quieter delegates who might otherwise be hesitant to contribute for the first time.

The action planning at the end of the course is essential to ensure that the learning is implemented [16, 17]. The 2 week time target is necessary for practical skills, because if left for longer the trainer may never initiate it, or may feel uncomfortable when doing so. The 2 month time target is appropriate for skills that may require the design of a form, the introduction of a process, or the influencing of colleagues. The 2 year time target gives a vision of the future which adds relevance to the shorter term actions. It is also more appropriate for wider scale targets, such as those requiring action at national level.

### Design and roll out of the TTT programme

The long term objectives of the programme as a whole were not drawn up until after the first course to ensure that they were appropriate to the needs of COECSA and African training [16, 17]. A key part of the programme was not only to teach trainers how to deliver ophthalmology training, but also to develop a faculty who could cascade the TTT Programme to the regions.

A COECSA TTT Lead was appointed after the first course and has been a vital link in communication and planning with the RCOphth TTT Lead. It is this close 1:1 relationship that has ensured that the programme has remained responsive to the varied and ever-changing needs of training throughout the region. She has also been essential in the identification of individuals suitable for promotion to Facilitator and Faculty Member.

It is valuable to have multiple stages of development of the faculty. Facilitators assist in the small group work enabling them to attend the course on multiple occasions to achieve spiral learning, and it also develops their confidence in discussing training issues and giving feedback to others. The Faculty Members can lead different sections each time they attend a course, collecting the experience which will enable them to deliver a half-day symposium or longer. The faculty are specifically taught facilitation skills, and are mentored with personalized constructive feedback.

The end of the first two-year grant came at a time when the African Faculty still lacked the critical mass and experience to be able to carry the project forwards alone. In order to prevent the decay of the existing achievements, it was essential to be able to continue the programme with minimal finance. Therefore short courses of half or one day, lead by a single UK trainer (the RCOphth TTT Lead) were attached to other events such as the Congress and the examination. The most valuable practicals from the three day course were delivered with minimal theory, and with assistance from the African faculty. The result was highly efficient courses at relatively low cost, which was beneficial to both the delegates and faculty development. Following the success of the half-day course preceding the Congress in 2015, the following year the course was given a nearly-full day in the main Congress programme. This was open to any Congress attendee, so attracted others with a real interest in training. These short courses kept the programme running, which was essential for obtaining the next 3-year grant, which is starting in 2017 enabling full roll-out to begin.

### Sustainability of the TTT programme

The COECSA TTT database lists all participants so far and the roles they have held. It also identifies a potential TTT Lead, Faculty Member and Facilitator as a minimum for each country, who will be invited to take up the role. The job descriptions detail the responsibilities they have for rolling out the programme in their own country, and how they should support each other.

The faculty will be invited to COECSA TTT courses where they will deliver material and be mentored, and well as receive training in more advanced topics. They will be given all the presentation and skills materials, e-learning and instructions on how to deliver the practicals. The national teams will then be able to roll out the TTT locally to colleague trainers, trainees and trainers in other professions.

Now that the COECSA College and its Faculty have seen the benefit of the training skills, they want to develop a COECSA Training System comprising the necessary tools and processes which will be designed during workshops before or after the courses. This will enable them to measure the quality of their trainees, trainers and units, to ensure that adequate standards are being met.

The extra funding for years 5–7 of the project are essential for embedding the necessary skills and processes throughout the region, and ensuring the outcome is sustainable [16]. By 2020 it is expected that the UK TTT Lead will be able to support from the UK without the need to visit, and that even this requirement will reduce fairly rapidly. Thereafter the enthusiasm of the COECSA TTT Lead will determine how the project continues to develop. For example, they could follow the UK model, to deliver courses of different levels (Advanced Trainers and/or Trainees), to different professions (nurses and OCOs), or to support other medical specialties.

### Benefits of the partnership to the UK

The reciprocal benefits of overseas partnerships to all the individuals concerned are well recognised to include a widening of cultural experience [16, 17]. A close relationship was rapidly built up between the two TTT Leads, and over a series of courses all the delegates and faculty felt like a team developing together, with a common purpose to improve their understanding of training and develop better skills and processes.

An additional personal benefit is the rejuvenation stimulated by working in a different environment on a project with hugely positive outcomes. Variety enriches one’s career, particularly at a time when there are massive clinical pressures in the National Health Service (NHS).

Discussing the different ways in which training was delivered in all the African institutions generated a variety of models, each with their own strengths and weaknesses. This stimulated reflection on the UK training scheme, and provided a pool of ideas which can be drawn on for the development of training in the UK and elsewhere.

Similarly, observation of the social interactions during the practical skills and discussions demonstrated new ways of handling these conversations. It also lead to a greater understanding of the behaviour of those from other backgrounds, which may also be valuable in the UK.

In evolution, the greater the environmental changes and pressures, the faster an organism adapts and develops. In the UK the TTT courses are being challenged by trainers being given less time away from clinical work to attend or deliver courses, and there is less funding available to pay for courses or travel expenses. Similar but greater challenges in Africa stimulated re-assessment of which parts of the course are most valuable and how to deliver them most efficiently. As a result, half- and one-day courses in the UK as well as Africa concentrate on the practicals for running a teaching session, leading effective feedback and using appraisal to support personal development. Both colleges have used the model of running courses along side or at other events, and now in the UK there is an annual half-day TTT symposium during RCOphth Annual Congress for 72 delegates using 12 facilitators and 2 leads.

Following the development of the cascade concept for Africa, a similar mechanism is being set up in the UK. A larger faculty is being trained and shortly their regional distribution will be analysed so more faculty can be recruited to gaps, and courses can be delivered locally across the whole country.

In Africa, eye care relies heavily on the multiprofessional team, and now RCOphth is leading on developing a model of multiprofessional service delivery and training in the UK [19].

### Impact of the TTT programme in Africa

The training of trainees and their trainers is used the world over on the assumption that teaching trainers to train improves the delivery of training [12], which in turn produces a more skilled workforce, and that is reflected in improved patient care. However, it is very difficult to accurately measure the impact of a programme such as Training the Trainers [3].

A follow up questionnaire completed by the delegates showed that they had implemented skills learned on the course, and were still utilizing them months and years later. Studies have shown that specific training techniques are effective, for example, Peyton’s four step technique for teaching a practical skill [13], and Pendleton’s four step method for giving feedback [14]. The assumption that better training results in more competent trainees is supported by the reduction in the length of the UK ophthalmology training programme from 10 to 14 years to 7 years [11].

It is also assumed that a well-trained trainee is more likely to deliver better patient care which will improve patient outcomes [3]. However in such a complex, evolving environment as the health care system in Sub-Saharan Africa, it is impossible to measure the patient impact of this programme, as distinct from other initiatives.

The programme also has wider impact which is difficult to measure, because good supervision supports the general professional and personal development of all trainees, and maximizes their potential, with far reaching effects. It also teaches generic skills that are important for communication, team working and leadership [20]. The associated changes in ethos can also have benefits across the whole eye care network, and spread to other disciplines.

The estimates of the number of patients managed by eye care workers who had been taught or supervised by someone who had benefitted directly or indirectly from the cascade of TTT, showed how the impact is potentially huge, at approaching 1 million ophthalmology patients per year. This is after only the first two years of the course, before the cascade has been initiated, so the effect is likely to be even greater in the future. This will be further amplified if the programme can be rolled out to other healthcare professions and specialties.

The next phase of the programme includes designing the tools and processes necessary to deliver the COECSA Training System. They will be used to assess the competency of trainees, the training abilities of trainers and the suitability of the units for training. Through these it will become easier to measure the direct outcomes of training, and identify areas for sharing good practice or targeting improvements.

When future public health surveys [3] document improved eye health within the CEOCSA Region, it is highly likely that the COECSA-RCOphth Training the Trainers Programme has contributed to this.

## Conclusions

The COECSA-RCOphth Training the Trainers Programme is a model of human resources development which can be applied to a wide variety of roles, specialties, healthcare professions and geographical locations. It cascades specific skills and processes through a pyramid of trainers within a healthcare network spread across several countries. This model delivers successful outcomes with a potentially broad impact on eye health, and ensures sustainability after completion of partnership support. The Programme also has significant benefits for the UK partners and organisations involved.

The COECSA-RCOphth Partnership was successful because of well-aligned aims and pre-existing links between the two organisations. A close one-to-one relationship between their two TTT Leads fostering discussion ensured that experienced trainers tailored the courses to the local situation, and that there was good engagement from the delegates.

The TTT Courses were highly effective because they fostered learning, practice and the implementation of skills. Rather than just imparting knowledge, they were able to change ethos and behaviour. Set preparatory and consolidation work for delegates before and after the course creates time for the face-to-face learning of practical skills, and supports spiral learning. The first session of the course sharing aims and objectives sets an interactive environment and helps with tailoring the course and setting expectations. Formative feedback during practical work ensures that delegates fulfill their potential, and objective evaluation of the course supports development of the programme. The last session of the course in which everyone shares their action plan with time scales, improves implementation rates.

Long-term sustainability of the Programme is dependent upon the cascade of skills from the UK Faculty through the COECSA Faculty Members and Facilitators to the delegates and beyond. On each course there is as much effort invested in the development of Faculty as there are the delegates. The COECSA Faculty should be given opportunities for the delivery of material and the facilitation of small group work, whilst being supported and mentored by the UK faculty. Every opportunity should be taken to promote the skills and ethos learnt on the courses, to reach more delegates and to integrate the new learning into existing events and structures.

The Programme has personal benefit to the UK Faculty through the cultural experience of working with a wide group of overseas delegates, and closely with the COECSA TTT Lead. Rejuvenation of their own career is stimulated by working in a different environment on a project with hugely positive outcomes. The institutional benefits to the RCOphth include exposure of its faculty to different training models and increased cultural awareness when delivering UK courses. The need to respond to the greater challenges in Africa has facilitated a faster evolution and diversification of courses in the UK.

When supported by the triad of partnership, effective training and cascade of skills, Programmes such as the COECSA-RCOphth TTT can become sustainable so they help develop a skilled motivated workforce who can deliver high quality healthcare.
